# Toward a comprehensive and realistic risk evaluation of engineered nanomaterials in the urban water system

**DOI:** 10.3389/fchem.2014.00039

**Published:** 2014-06-23

**Authors:** Lars Duester, Michael Burkhardt, Arno C. Gutleb, Ralf Kaegi, Ailbhe Macken, Björn Meermann, Frank von der Kammer

**Affiliations:** ^1^Department G2 - Aquatic Chemistry, Federal Institute of Hydrology, BfGKoblenz, Germany; ^2^Institute of Environmental and Process Engineering (UMTEC), HSR University of Applied SciencesRapperswil, Switzerland; ^3^Département Environnement et Agro-biotechnologies (EVA), Centre de Recherche Public - Gabriel LippmannBelvaux, Luxembourg; ^4^Department of Process Engineering, Eawag, Swiss Federal Institute of Aquatic Science and TechnologyDuebendorf, Switzerland; ^5^Ecotoxicology and Risk Assessment, Norwegian Institute for Water ResearchOslo, Norway; ^6^Department of Environmental Geosciences, University of ViennaVienna, Austria

**Keywords:** engineered nanomaterials, water treatment, wastewater, urban water system, fate of nanomaterials, nanomaterials toxicity, transformation of nanomaterials, risk assessment on nanomaterials

## Abstract

The European COoperation in Science and Technology (COST) Action ES1205 on the transfer of Engineered Nano materials from wastewater Treatment and stormwatEr to Rivers (ENTER) aims to create and to maintain a trans European network among scientists. This perspective article delivers a brief overview on the *status quo* at the beginning of the project by addressing the following aspects on engineered nano materials (ENMs) in the urban systems: (1) ENMs that need to be considered on a European level; (2) uncertainties on production-volume estimations; (3) fate of selected ENMs during waste water transport and treatment; (4) analytical strategies for ENM analysis; (5) ecotoxicity of ENMs, and (6) future needs. These six step stones deliver the derivation of the position of the ES1205 network at the beginning of the projects runtime, by defining six fundamental aspects that should be considered in future discussions on risk evaluation of ENMs in urban water systems.

## Introduction

**Figure 1:**
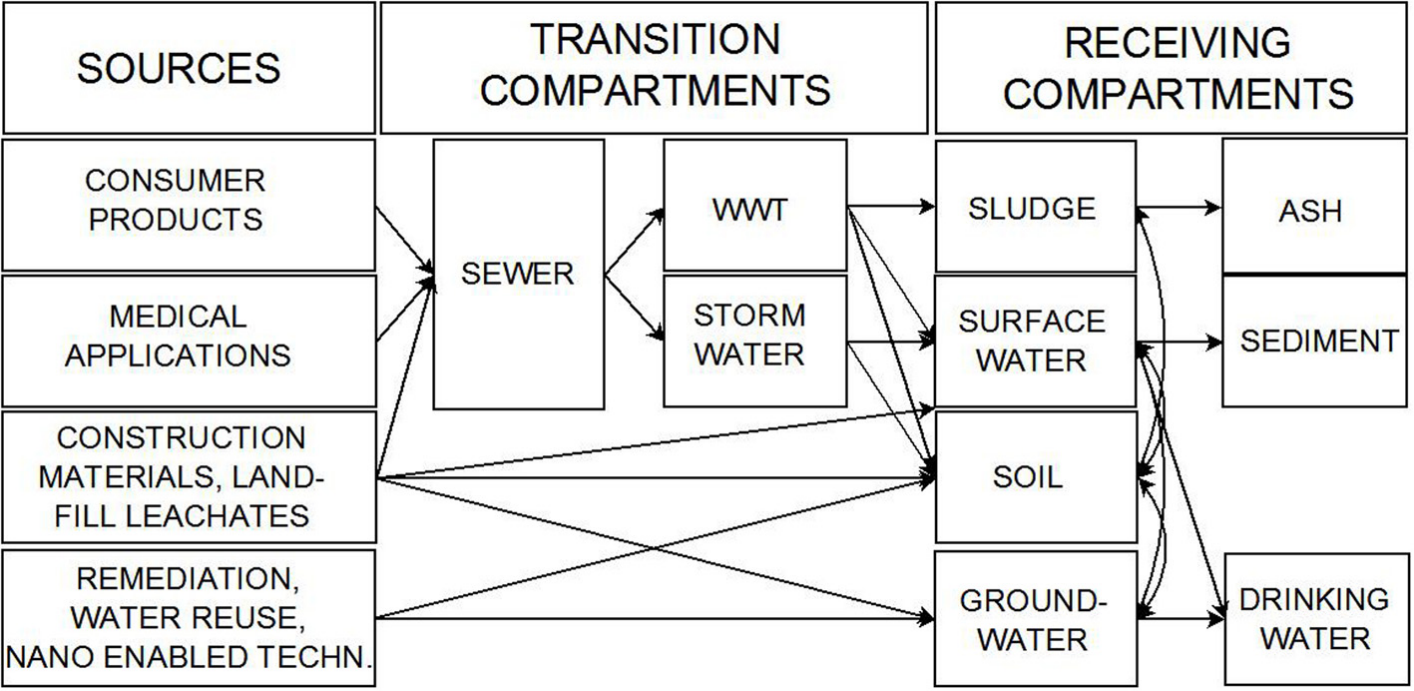
**Schematic overview on different potential pathways of ENMs focusing on the urban water system**.

The COST Action ES1205 ENTER was launched in June 2013 (http://www.es1205.eu and http://www.cost.eu/domains_actions/essem/Actions/ES1205). In close cooperation with the NORMAN network working group 4 (http://www.norman-network.net/?q=node/54) the Action aims to create and maintain a lasting pan European network among scientists to gain a better understanding of the role of the urban water systems controlling the release of ENMs to the aquatic and associated environments (e.g., sewage sludge or wetland soils, and sediments). By May 2014 28 COST and none COST countries as well as more than 140 scientists joined the network. Key questions are which and what amounts of ENMs are released, how persistent are they and to what extent do they cause *in situ* toxicity? The scope of this perspective article aims to sum up the status and lack of current knowledge in answering the above mentioned questions. Out of several sources-sink scenarios (Figure 1) the COST Action ES1205 will prioritize the potential pathways in the following order:

1. wastewater → wastewater treatment (WWT) → surface water, sediments;
2. wastewater → sewage sludge → (incineration plant) ash disposal, soil conditioner, fertilizer → soil;
3. wastewater → stormwater → surface water, sediments, stormwater treatment systems (e.g., constructed wetlands), and
4. landfill leachate → treatment → surface water, sediments.

As soon as the step from fundamental research toward considerations on the environmental relevance of ENMs is undertaken, the production volumes of ENMs have to be evaluated and their mobility in the environment has to be assessed. Different release, exposure and fate scenarios have to be evaluated, depending on the application and life cycle of different types of ENMs. Consequently, a connection between the ENM applications and potential sources into urban water systems has to be established.

A thorough understanding of potential pathways and linkage of transformation and retention processes of ENMs in different compartments is highly needed to enable a valid risk evaluation. A fundamental requirement for a mechanistic understanding of ENM behavior in addition to ecotoxicity studies, are sufficiently applicable analytical tools for complex matrices. This, in connection with fate and toxicity studies enables a comprehensive and realistic risk evaluation of ENMs in urban water systems.

This perspective article highlights and critically discusses briefly the *status quo* of scientific findings regarding ENMs at the beginning of the COST Action. Given the fact that silver nanoparticles (Ag-NPs) are strongly discussed in science and the public (but of less relevance from an application and transformation point of view) literature was cited (where possible) for clarity reasons.

## Considered ENMs on an european level

ENMs cover a heterogeneous range of different materials, including: inorganic (non-metallic), carbon based, metal, and organic nanomaterials. Due to this and the fact that a uniform statutory demanded product labeling, indicating whether “nano” is “inside” a product or not, is mostly voluntary, market information on ENM production volumes and types are not readily available.

Hence, the European Commission (EC) composed a working paper on types and uses of nanomaterials (EU Commission, 2012) providing an overview on ENMs commercially in use. On the basis of this working paper it turned out that two materials in the nano range dominate the market: carbon black (9.6 million t/a) and amorphous silica (1.5 million t/a). Further ENMs produced in significant amounts are: aluminium oxide (200 kt/a), barium titanate (15 kt/a), titanium dioxide (10 kt/a), cerium oxide (10 kt/a), zinc oxide (8 kt/a), and carbon materials (bandwidth 0.1–1 kt/a). Silver has been listed with minor priority (20 t/a). Main fields of applications of the above listed ENMs are: reinforcing agents in rubber goods (mainly carbon black, amorphous silica) followed by applications in electronics (e.g., fine abrasives, capacitors), cosmetics, biomedical applications (e.g., gold for diagnostics, silver in textiles), paints (e.g., pigments) and surface-coatings (e.g., scratch-resistance). Nevertheless, assessments on market information of ENMs need to be taken with caution.

As a further source of information a number of web-based platforms exist listing ENMs being commercially used. This data is linked to “day-to-day use goods” (e.g., at http://www.nanotechproject.org/ or www.nanowerk.com). But again, this information needs to be interpreted with caution. As nanotechnology is a rapidly developing field opening up new applications and markets, future trends need to be considered as well (“second,” “third,” and “fourth” generation materials, cf. EU Commission, 2012). This task is also addressed at, e.g., the recently started FP7 project FutureNanoNeeds attempting to deliver an overview on recent trends for next generation materials that are not on the market yet (www.futurenanoneeds.eu/). However, to derive a prioritization on potential “relevant” ENMs and their release pathways to be addressed in ES1205, additional information is needed on production and application volumes.

## Production-volume and transfer pathways into the urban water system

As a result of the lack of exposure data to receiving compartments, an assessment of release of ENMs into the environment by modeling the mass flow is currently the method of choice (e.g., Blaser et al., 2008; Mueller and Nowack, 2008; Petersen et al., 2011). Recent studies compared several peer reviewed publications and addressed the mass flow of ENMs into different compartments on a global level (e.g., Gottschalk et al., 2013; Keller et al., 2013). Most authors pronounce that the major source of modeling error is related to the uncertainty of production and application. Key input quantities, beside those mentioned in the previous section, are information from industry independent institutions, market surveys and interviews on production volumes, patents, and publications all related to limited insight to real market products. Hence, major challenges are associated with production and application information that are more representative than the current data available. Nevertheless, all of the authors cited in this paragraph seem to agree that the studies help to receive “bandwidth” information on expected environmental concentrations. This approach might be significantly improved, if the models are fed with more reliable input data.

But, how realistic is an improvement of the reliability of data on production volumes and applications? There are several factors that may interfere with the release of more detailed information, e.g., (1) an insufficient trust-based relationship between “public founded science” and “industry,” (2) market competition interests of individual producers, (3) worries about an “over critical” public perception of these numbers or in contrast to this (4) a gap between industrial “nano” promises and socioeconomic innovation payback from “day-to-day” applications, which becomes visible by detailed numbers.

Independently from the aforementioned reasons, this bottleneck can be considered as one of the most significant open issue when the environmental relevance or the potential impact (defined here as occurrence in the environment combined with *in situ* toxicity) of ENMs is discussed. In addition, challenges on definitions and standardization further complicate this situation.

## Transformation and retention processes during sewage passage/treatment

Taking the results from Ag-NP spiking experiments in a combined sewer system (Kaegi et al., 2013) as an example, a very efficient transport of ENMs along the sewer channel, with a significant transformation of the particles, can be expected. Electron microscopic investigation revealed that Ag-NP were very quickly attached to the sewer biomass and transported as “complex colloids” along the sewer line underlining the importance of heteroaggregation, especially at high ionic strength and suspended solid content in wastewater. Thus, waste water transport and treatment facilities are major hubs controlling the release of ENMs to the environment. However, also in cities with a state of the art sewer infrastructure a fraction of the ENMs will be directly discharged to surface waters or to treatment facilities (e.g., constructed wetlands) through the stormwater overflow during heavy rain events.

The removal of different materials, including Ag-NP (Kaegi et al., 2011; Doolette et al., 2013; Li et al., 2013), ZnO (Ma et al., 2013), TiO_2_ (Westerhoff et al., 2011), CeO_2_ (Limbach et al., 2008) in wastewater treatment plants (WWTPs, mostly activated sludge processes) have been studied in detail, and consistently very high removal efficiencies of approximately 95% or more have been reported. Therefore, ENMs are very efficiently removed from the wastewater stream and accumulated in the sludge biomass during the wastewater treatment. Consequently, at the moment, low Ag-NP concentrations in the effluent are reported [order of magnitude: ng/L (Li et al., 2013)] and released to the surface water.

The agricultural application of sewage sludge as fertilizer may result in a release of the ENMs accumulated in the sludge to the soil and to surface water as well as to the groundwater. Whether increased loads of ENMs from the sewage sludge have any negative effect on soil properties, or whether the ENM from sewage sludge are increasingly taken up by plants is not fully understood, yet.

As an example, in urban wastewater systems the transformation of Ag-NP to silver sulfide (Ag_2_S) is of prime interest due to dramatic reduction of the toxicity of the Ag-NP already after partial sulfidation (Reinsch et al., 2012). The sulfidation process already starts in the sewer system (Kaegi et al., 2013) which is readily explained by considerable concentration of bisulfide reported from sewer systems (Nielsen et al., 2008). Near complete sulfidation has been observed in the WWTP (activated sludge process) and also in digested sludge the speciation of Ag is strongly dominate by Ag_2_S (Doolette et al., 2013; Lombi et al., 2013; Ma et al., 2013). These findings are consistent with the observation of nanoscale Ag_2_S in sewage sludge collected from full scale WWTP (Kim et al., 2010). Ag_2_S is very resistant toward oxidation with O_2_ and only minor fraction of Ag^+^ have been released from Ag_2_S over periods of several months (Levard et al., 2011; Lombi et al., 2013). To follow these changes in speciation/fractionation of ENMs in wastewater, sludge, soils and sediments sophisticated analytical techniques have to be improved.

## Analytical technique evaluation: challenges and performances

The EC recommendation for a definition of ENMs (EU Commission, 2011) asks for number-based particle size distributions to classify a material as nano or not. However, this material classification strategy should not be applied equally to all sectors, e.g., environmental monitoring of NPs, where other properties than particle-number concentrations reveal importance.

In general an analytical strategy must be adapted to the analyte, the required information and a given matrix (von der Kammer et al., 2012) but in terms of ENMs in (urban) water systems a variety of analytical challenges need to be tackled: (1) to-be-expected low mass concentrations, (2) the presence of natural and incidental particles of similar size, shape, and composition, (3) dissolution, agglomeration, association (e.g., natural colloids, cells, proteins) and transformation of ENMs, as well as (4) a lack of certified reference materials (CRMs) allowing for method validation.

Regarding inorganic ENMs with a relatively high natural background concentration of certain elements (e.g., SiO_2_, TiO_2_, ZnO) or an interfering proportion of similar incidental particles (e.g., Pt and Pd from automotive catalysts), the differentiation from natural background or incidental particles is challenging. It might be achieved by, e.g., elemental composition and/or isotopic-ratio determination (Weber et al., 2009; Tuoriniemi et al., 2012), using specific impurities in the inorganic ENMs or in the particles of the natural background [e.g., La in natural CeO_2_ (von der Kammer et al., 2012)] or would only be possible on a single-particle based analysis using electron microscopy or single particle inductively coupled plasma-mass spectrometry (Pace et al., 2012; Tuoriniemi et al., 2012). Referring to ENMs with vanishing particulate background concentrations [e.g., Au, Ag, although they might be formed naturally under certain conditions (Weber et al., 2009)], a direct measurement of particulate concentrations might be sufficient in cases when interference from natural sources are unlikely.

Next to analytical technical challenges, CRMs are mostly still missing—comprehensive method validation becomes difficult and alternative strategies need to be developed in their absence. The parts of an analytical method being most error-prone are sampling and sample preparation. Standard-operating procedures (SOPs) addressing the sample preparation and analysis for most relevant ENMs are missing.

Although the number of available analytical methods is increasing, method development is still challenging, especially in terms of environmental samples and toxicity data verification. Furthermore, method validation and result assessment is made difficult due to missing SOPs and CRMs. In our opinion cost-efficient and straightforward applicable analytical methods are needed providing valid results to evaluate whether ENMs are present in a given sample and at which concentration. A crucial prerequisite to reach this goal are “nano” CRMs rectified in size and fraction-related quantity which are still lacking.

## Ecotoxicity-based considerations

In recent years a large amount of time and effort as well as funding opportunities have focused on the investigation into the fate and effects of ENMs on human health and the environment. Although this research is novel and bespoke it is not necessarily the most useful when considering regulations and risk assessment (RA). There is also a lack of comparative studies with bulk products in order to identify possible nano-specific effects and assess the need for nano-specific regulations. Therefore, a lot of data available in the literature may not be applicable in the decision-making process on the need for nano-specific regulatory requirements or recommendations. In general, there is a consensus view that existing regulations should suffice.

The RA paradigm (hazard identification and characterization followed by exposure assessment and risk characterization) is considered appropriate (EFSA Scientific Committee, 2011). However, there may be a need for more specific guidance on certain aspects of their assessment. Toxicity often cannot be related to the actual size, the mass or the surface area of the single NPs or the NP agglomerates in exposure media even under standardized laboratory conditions. Therefore, it can be proposed that the toxicity is greatly influenced by other inherent and poorly understood properties of the particles that are not considered in standard guidance documents. While for *in vitro* studies the necessity to study interactions of ENMs and proteins but also other molecules in the exposure medium is of crucial importance in order to understand observed effects (Lynch et al., 2013) this is not yet commonly considered in studies using aquatic invertebrates. Important aspects that will need careful consideration in the future are the interactions of ENMs within the environmental compartments and combined effects with legacy chemicals (e.g., Farkas et al., 2012), correct dosimetry, formulation, and exposure route to organisms, characterization, and transformation in the environment (e.g., Ag-NPs: Impellitteri et al., 2013; Lombi et al., 2013) but also the possibility of food chain effects (e.g., Shu et al., 2010). All of which are difficult to analyse and quantify, therefore (along with the challenges regarding input quantities and analyses) making a realistic RA nearly impossible at the present time.

There is a need for nano predicted effect/no effect concentrations (PEC/PNECs) for receiving environmental compartments which form an essential part of RA (EU Commission, 2003; ECHA, 2008). Simple models to derive PECs and to address likely sinks need to be developed to compensate for the uncertainties in the data available on aspects of ENMs such as usage, concentrations (in products) and release into the environment and thereby support the generated ecotoxicity data leading to better and more realistic RA of this class of contaminants.

Despite a growing opinion that existing measures are suitable for the hazard and risk characterization of ENMs, nano risk tends to be treated on a case-by-case basis and this will not be sustainable in the future as more and more novel materials and products come on the market. The scientific community is discussing but not yet ready to answer how to deal with new biological mechanisms and processes arising from novel nanostructures, eco-identity, Trojan-horse effect, etc. and how to make clear cut decisions on regulations.

## Critical appraisal

To better coordinate and to structure scientific endeavors on ENM RA the following points should be considered in future evaluations:

1. For all ENMs studied so far, a removal efficiency >95% in WWTPs was shown. As a consequence sewage sludge pathways should be addressed.
2. In urban wastewater ENMs undergo different transformations reactions which generally reduce the reactivity. Hence, the properties and fate of transformed ENMs with regard to speciation and fractionation need to be addressed.
3. Studies on remobilization/speciation of ENMs from/in ashes, sediments, landfills, and soils are mostly lacking, this gap has to be addressed within the next years.
4. There is a challenge to differentiate between good ecotoxicity studies with, e.g., sufficient analytical verification and a high informative value and those that lack quality as well as environmental relevance. Most of the studies are effect and not mechanism focused. Studies that allow statements on *in situ* effects are lacking. These scientific drawbacks can be addressed by defining, e.g., basic quality criteria for studies with ENM.
5. Analytical methods tailored to selectively detect ENMs are important to study the interaction of ENMs understand mechanisms and processes (e.g., cell wall/membrane transfer of ENMs). Furthermore, “nano” CRMs certified in size and fraction related quantities are highly needed for method validation. For monitoring initiatives and ecotoxicity studies, the analytical methods including sample preparation protocols need to be easily applicable to complex matrices and operationally defined extraction methods [e.g., enrichment via cloud point extraction combined with atomic absorption spectroscopy (Hartmann and Schuster, 2013)], may help to allow for distinction between dissolved species and particles, if verified by other methods (Fabricius et al., 2014). As an example for Ag-NPs at the moment, total Ag content analyses seem appropriate for surface and groundwater monitoring. In future activities it will be very important to evaluate which ecotoxic effects can be expected if the total amount of Ag < 0.45 μm equals 100, 50, or 10% ENM and how the degree of transformation of the ENMs modulates the expected ecotoxicity. This may raise the need to derive nano specific PNECs and to compare these with existing PNECs of the receiving water bodies.
6. The access to data on production volumes and applications is poor. This is a major missing link for the prioritization of ENMs in environmental RAs and can only be counteracted by an improved trust-based relation between industries and scientists or a labeling of “nano” products, which show the potential to release a “relevant” nano fraction. However, this is a discussion connected to many different challenges and driven by many different interests.

Although surface waters represent one of the most important receiving compartment for ENMs, based on the available scientific knowledge the authors representing the COST Action ES1205 ENTER do not see, at the moment, a general need for establishing nano-specific monitoring programs for surface waters. However, surface waters in the vicinity of production facilities may require increased attention.

### Conflict of interest statement

The authors declare that the research was conducted in the absence of any commercial or financial relationships that could be construed as a potential conflict of interest.

## References

[B1] Blaser S. A. Scheringer M. Macleod M. Hungerbuhler K. (2008). Estimation of cumulative aquatic exposure and risk due to silver: contribution of nano-functionalized plastics and textiles. Sci. Total Environ. 390, 396–409 10.1016/j.scitotenv.2007.10.010 18031795

[B2] Doolette C. L. McLaughlin M. J. Kirby J. K. Batstone D. J. Harris H. H. Ge H. (2013). Transformation of PVP coated silver nanoparticles in a simulated wastewater treatment process and the effect on microbial communities. Chem. Cent. J. 7, 46 10.1186/1752-153X-7-46 23497481 PMC3636095

[B3] ECHA. (2008). Guidance On Information Requirements and Chemical Safety Assessment. Chapter r.7b: Endpoint Specific Guidance. (Version 1.2). Guidance for The Implementation of REACH. Available online at: http://echa.europa.eu/documents/10162/13632/information_requirements_r7b_en.pdf

[B4] EFSA Scientific Committee. (2011). Scientific Opinion on Guidance on the risk assessment of the application of nanoscience and nanotechnologies in the food and feed chain. EFSA J. 9:2140 10.2903/j.efsa.2011.2140 32625968 PMC7009542

[B5] EU Commission. (2003). Technical Guidance Document on Risk Assessment. Institute for Health and Consumer Protection, European Chemicals Bureau. Part II. Available online at: http://echa.europa.eu/documents/10162/16960216/tgdpart2_2ed_en.pdf

[B6] EU Commission. (2011). Commission Recommendation of 18 October 2011 On The Definition of Nanomaterial (2011/696/EU). Official Journal of the European Communities: Legis

[B7] EU Commission. (2012). Types and Uses of Nanomaterials, Including Safety Aspects (COM(2012) 572 Final). Available online at: http://eur-lex.europa.eu/LexUriServ/LexUriServ.do?uri=SWD:2012:0288:FIN:EN:PDF

[B8] Fabricius A.-L. Duester L. Meermann B. Ternes T. (2014). ICP-MS-based characterization of inorganic nanoparticles—sample preparation and off-line fractionation strategies. Anal. Bioanal. Chem. 406, 467–479 10.1007/s00216-013-7480-2 24292431 PMC3885803

[B9] Farkas J. Nizzetto L. Thomas K. V. (2012). The binding of phenanthrene to engineered silver and gold nanoparticles. Sci. Total Environ. 425, 283–288 10.1016/j.scitotenv.2012.03.010 22483949

[B10] Gottschalk F. Kost E. Nowack B. (2013). Engineered nanomaterials in water and soils: a risk quantification based on probabilistic exposure and effect modeling. Environ. Toxicol. Chem. 32, 1278–1287 10.1002/etc.2177 23418073

[B11] Hartmann G. Schuster M. (2013). Species selective preconcentration and quantification of gold nanoparticles using cloud point extraction and electrothermal atomic absorption spectrometry. Anal. Chim. Acta 761, 27–33 10.1016/j.aca.2012.11.050 23312311

[B12] Impellitteri C. A. Harmon S. Silva R. G. Miller B. W. Scheckel K. G. Luxton T. P. (2013). Transformation of silver nanoparticles in fresh, aged, and incinerated biosolids. Water Res. 47, 3878–3886 10.1016/j.watres.2012.12.041 23561507

[B13] Kaegi R. Voegelin A. Ort C. Sinnet B. Thalmann B. Krismer J. (2013). Fate and transformation of silver nanoparticles in urban wastewater systems. Water Res. 47, 3866–3877 10.1016/j.watres.2012.11.060 23571111

[B14] Kaegi R. Voegelin A. Sinnet B. Zuleeg S. Hagendorfer H. Burkhardt M. (2011). Behavior of metallic silver nanoparticles in a pilot wastewater treatment plant. Environ. Sci. Technol. 45, 3902–3908 10.1021/es1041892 21466186

[B15] Keller A. A. McFerran S. Lazareva A. Suh S. (2013). Global life cycle releases of engineered nanomaterials. J. Nanopart. Res. 15, 6 10.1007/s11051-013-1692-4

[B16] Kim B. Park C.-S. Murayama M. Hochella M. F. (2010). Discovery and characterization of silver sulfide nanoparticles in final sewage sludge products. Environ. Sci. Technol. 44, 7509–7514 10.1021/es101565j 20839838

[B17] Levard C. Reinsch B. C. Michel F. M. Oumahi C. Lowry G. V. Brown G. E. (2011). Sulfidation processes of PVP-coated silver nanoparticles in aqueous solution: impact on dissolution rate. Environ. Sci. Technol. 45, 5260–5266 10.1021/es2007758 21598969

[B18] Li L. Hartmann G. Doblinger M. Schuster M. (2013). Quantification of nanoscale silver particles removal and release from municipal wastewater treatment plants in Germany. Environ. Sci. Technol. 47, 7317–7323 10.1021/es3041658 23750458

[B19] Limbach L. K. Bereiter R. Muller E. Krebs R. Galli R. Stark W.J. (2008). Removal of oxide nanoparticles in a model wastewater treatment plant: influence of agglomeration and surfactants on clearing efficiency. Environ. Sci. Technol. 42, 5828–5833 10.1021/es800091f 18754516

[B20] Lombi E. Donner E. Taheri S. Tavakkoli E. Jaemting A. K. McClure S. (2013). Transformation of four silver/silver chloride nanoparticles during anaerobic treatment of wastewater and post-processing of sewage sludge. Environ. Pollut. 176, 193–197 10.1016/j.envpol.2013.01.029 23434771

[B21] Lynch I. Ahluwalia A. Boraschi D. Byrne H. J. Fadeel B. Gehr P. (2013). The bio-nano-interface in predicting nanoparticle fate and behaviour in living organisms: towards grouping and categorising nanomaterials and ensuring nanosafety by design. BioNanoMaterials 14, 195–216 10.1515/bnm-2013-0011

[B22] Ma R. Levard C. Judy J. D. Unrine J. M. Durenkamp M. Martin B. (2013). Fate of zinc oxide and silver nanoparticles in a pilot wastewater treatment plant and in processed biosolids. Environ. Sci. Technol. 48, 104–112 10.1021/es403646x 24266610

[B23] Mueller N. C. Nowack B. (2008). Exposure modeling of engineered nanoparticles in the environment. Environ. Sci. Technol. 42, 4447–4453 10.1021/es7029637 18605569

[B24] Nielsen A. H. Vollertsen J. Jensen H. S. Madsen H. I. Hvitved-Jacobsen T. (2008). Aerobic and anaerobic transformations of sulfide in a sewer system - Field study and model simulations. Water Environ. Res. 80, 16–25 10.2175/106143007X184537 18254394

[B25] Pace H. E. Rogers N. J. Jarolimek C. Coleman V. A. Gray E. P. Higgins C. P. (2012). Single particle inductively coupled plasma-mass spectrometry: a performance evaluation and method comparison in the determination of nanoparticle size. Environ. Sci. Technol. 46, 12272–12280 10.1021/es301787d 22780106

[B26] Petersen E. J. Zhang L. Mattison N. T. O'Carroll D. M. Whelton A. J. Uddin N. (2011). Potential release pathways, environmental fate, and ecological risks of carbon nanotubes. Environ. Sci. Technol. 45, 9837–9856 10.1021/es201579y 21988187

[B27] Reinsch B. C. Levard C. Li Z. Ma R. Wise A. Gregory K. B. (2012). Sulfidation of silver nanoparticles decreases escherichia coli growth inhibition. Environ. Sci. Technol. 46, 6992–7000 10.1021/es203732x 22296331

[B28] Shu X. Wang J. Zhang X. Chang Y. Chen Y. (2010). Trophic transfer of TiO_2_ nanoparticles from daphnia to zebrafish in a simplified freshwater food chain. Chemosphere 79, 928–933 10.1016/j.chemosphere.2010.03.022 20371096

[B29] Tuoriniemi J. Cornelis G. Hassellov M. (2012). Size Discrimination and detection capabilities of single-particle ICPMS for environmental analysis of silver nanoparticles. Anal. Chem. 84, 3965–3972 10.1021/ac203005r 22483433

[B30] von der Kammer F. Ferguson P. L. Holden P. A. Masion A. Rogers K. R. Klaine S. J. (2012). Analysis of engineered nanomaterials in complex matrices (environment and biota): general considerations and conceptual case studies. Environ. Toxicol. Chem. 31, 32–49 10.1002/etc.723 22021021

[B31] Weber F. A. Voegelin A. Kaegi R. Kretzschmar R. (2009). Contaminant mobilization by metallic copper and metal sulphide colloids in flooded soil. Nat. Geosci. 2, 267–271 10.1038/ngeo476

[B32] Westerhoff P. Song G. Hristovski K. Kiser M. A. (2011). Occurrence and removal of titanium at full scale wastewater treatment plants: implications for TiO_2_nanomaterials. J. Environ. Monit. 13, 1195–1203 10.1039/c1em10017c 21494702

